# *Pantoea agglomerans* Bacteremia: Is It Dangerous?

**DOI:** 10.1155/2020/7890305

**Published:** 2020-04-03

**Authors:** Ikwinder Preet Kaur, Sindhura Inkollu, Amulya Prakash, Haresh Gandhi, Mohsin Sheraz Mughal, Doantrang Du

**Affiliations:** Monmouth Medical Center, Long Branch, NJ, USA

## Abstract

*Introduction*. *Pantoea agglomerans*, an anaerobic Gram-negative bacillus, is a rare cause of opportunistic infections affecting premature infants to seniors. We present a 34-year-old man who was presented for the management of diabetic ketoacidosis and developed *Pantoea agglomerans* bacteremia after one week of hospitalization. *Case Presentation*. A 34-year-old African-American male with uncontrolled diabetes mellitus type I and recurrent skin infections was admitted with diabetic ketoacidosis. He had left upper extremity abscess, preliminary wound cultures were positive for Gram-positive cocci, and an initial set of blood cultures were negative. He was started empirically on vancomycin. One week after admission, he started having chills followed by a recurrent increase in body temperature to 102 degrees Fahrenheit. The wound was healing, without active infection. Chest X-ray and CT scan of abdomen and pelvis to rule out infection were negative. Repeat blood cultures showed *P. agglomerans* in both the tubes. The patient was successfully treated with intravenous ceftriaxone, and he recovered fully without any complication. *Discussion*. *Pantoea agglomerans* is a bacteria associated with plants; however, it can infect humans and vertebrate animals. The outcome seems favourable with the institution of appropriate antibiotics even in immunocompromised patients.

## 1. Introduction

*P. agglomerans* is a rare cause of opportunistic human infections caused by wound infection with plant material or as a hospital-acquired infection mostly in immunocompromised individuals. It reveals a diverse clinical picture and can affect both immunocompetent and immunocompromised individuals with patient age ranging from premature infants to seniors [1]. Based on the scientific databases including PubMed and Google Scholar, we found that although bloodstream infections are often associated with contaminated medical equipment leading to outbreaks, mention of sporadic bacteremia in English literature is limited to a few case reports [2]. In this article, we are describing a case of sporadic *P. agglomerans* bacteremia in a middle-aged man presented with diabetic ketoacidosis (DKA).

## 2. Case Presentation

A 34-year-old African-American male with a history of uncontrolled diabetes mellitus type I and recurrent skin infections secondary to intravenous drug abuse was admitted with diabetic ketoacidosis (DKA) in September 2019 at Monmouth Medical Center, New Jersey, United States. His other history included but not limited to chronic pancreatitis, polysubstance abuse, multiple bacteremia episodes in past with MRSA, *Serratia marcescens* and *Pseudomonas*, untreated hepatitis B, and lung empyema status post right lung resection. On admission, vitals were stable. The patient was afebrile. Labs were consistent with DKA. A urine drug screen was positive for cocaine and opiates.

## 3. Hospital Course

**Figure 1:**
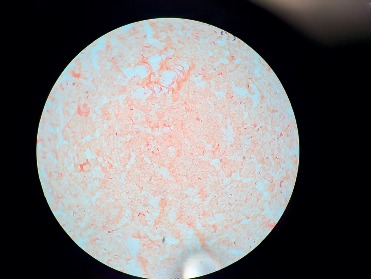
*Pantoea Agglomerans* on Gram stain (a Gram-negative bacillus).

**Figure 2:**
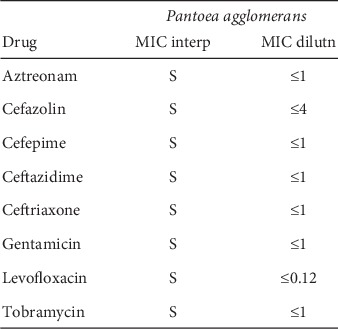
Antibiotic susceptibility of cultured *P. agglomerans*.

Patient was initially treated with intravenous (I.V.) insulin along with I.V. fluid boluses as per DKA treatment protocol, and the later was started on subcutaneous insulin. Electrolytes were replete appropriately. On the third day of hospitalization, he was found to have a subcutaneous soft tissue abscess in the medial aspect of left antecubital fossa measuring 2.6 × 1 × 2.4 cm. The abscess was incised and drained. Since preliminary wound cultures were positive for Gram-positive cocci, the patient was started empirically on vancomycin. An initial set of blood cultures were negative. One week after admission, the patient started having chills followed by an increase in body temperature to 102 degrees Fahrenheit, not relieved with antipyretics. The wound was healing, without active infection. White blood count was within normal limits. Chest X-ray was negative for any infiltrates/pneumonia/pleural effusion. CT scan of the abdomen did not show any abdominal abscess or source of infection. It showed the possibility of gastroesophageal reflux disease and gastroparesis secondary to opioids and DM. Repeated blood cultures initially showed Gram-negative rods in both tubes with Gram-positive cocci in pairs and chains in one tube. With the thought that bacteria might be resistant to vancomycin, antibiotics were switched to broad-spectrum with daptomycin to cover Gram-positive bacteremia and meropenem to cover Gram-negative bacteremia empirically. Final blood cultures showed *Pantoea agglomerans* in both the tubes (Figure 1) and leuconostoc citreum in one tube. Antibiotic susceptibility of cultured *P. agglomerans* is mentioned below (Figure 2). Meropenem was deescalated to ceftriaxone based on antimicrobial susceptibility and the patient was treated successfully with ceftriaxone while in hospital and was discharged with a prescription of levofloxacin. Repeat Blood cultures showed no bacterial growth.

## 4. Discussion

The bacterial genus *Pantoea* is a nonencapsulated, non-spore-forming anaerobic Gram-negative bacillus belongs to the family Enterobacteriaceae. It is associated with plants, however, can infect humans and vertebrate animals. It has approximately 20 species with *P. agglomerans*, previously known as *Enterobacter agglomerans* or *Erwinia herbicola* being the most prominent species in humans [3]. Clinical outcomes of infection caused by *Pantoea* includes infecting bones, joints, synovium causing septic arthritis, osteomyelitis, or synovitis [4] as very common to uncommon presentations including but not limited to endocarditis [5], endophthalmitis [6], and cutaneous infections. Cutaneous infections occur as a wound superinfection, or the organism may enter the skin when penetrating trauma occurs to the skin causing a chronic inflammatory response. The infection may extend deep into the bones to cause septic arthritis, spondylodiscitis, or tibial osteitis, and it may progress to peritonitis and sepsis [7]. Other than wound infection with plant material leading to above-mentioned outcomes, exposure to medical equipment or fluids contaminated with bacteria, especially in immune-compromised individuals is a major cause of infection which leads to bacteremia outbreaks. The factory-contaminated screw-caps of bottles with intravenous fluids is a proven example of an epidemic in the United States back in 1970-1971 in infants and children [8]. Other documented examples of iatrogenic outbreaks in pediatric patients include but not limited to septicemia with respiratory failure caused by parenteral nutrition solutions and septicemia with GI symptoms by contaminated transference tube used for intravenous rehydration [9]. In a study, the organism has been found as a potential candidate as powdered infant mild formula-borne opportunistic pathogen. [10]. In adults, examples of hospital-acquired *P. agglomerans* infection with an identified source of contamination include patients receiving hemodialysis or plasmapheresis, caused by contamination of an anticoagulant citrate-dextrose 46% solution [11], a and septicemia after blood transfusion [12].

Although bloodstream infections are often associated with contaminated intravascular products and medical equipment leading to an outbreak, *P. agglomerans* can cause spontaneous or sporadic bacteremia as well. There is a strong association of spontaneously occurring bacteremia with gastroesophageal reflux disease (GERD) and receipt of antacids. Since *Pantoea* species are commonly found on plants, there is a possibility of being introduced by ingestion of vegetables or fruits, with the opportunity for gastrointestinal (GI) translocation in the presence of gastroesophageal mucosal lesions as in GERD and/or in the absence of protective stomach acidity as with antacids [2]. Apart from transmission through ingestion of bacteria in food and direct penetration of human skin through microtrauma and/or medical devices as mentioned above, it can occur through occupational exposure to organic dust as well [13]. Other than GERD, spontaneous bacteremia has been associated with underlying pathologies like active malignancy, diabetes mellitus, chronic viral hepatitis, cerebrovascular accident, congestive heart failure, autoimmune or connective tissue diseases, chronic pulmonary obstructive disease, and end-stage renal disease. Also, ABO blood group A is more likely associated with *P. agglomerans* bacteremia, likely due to its well-known association with certain gastrointestinal disorders [2].

Bacterial endotoxin leads to cytokine production and is the responsible agent for infection. Signs and symptoms of bacteremia include but not limited to nonspecific pathophysiological reactions, such as gastrointestinal symptoms, fever, changes in white blood cell counts, anaemia, thrombocytopenia, disseminated intravascular coagulation, hypotension, and shock [13]. Diagnosis is usually made with positive cultures from specimens including blood, pus, urine, tracheal aspirate, and central venous line samples. With regards to effective antibacterial treatment, antimicrobial susceptibility was studied in a cohort study of spontaneous bacteremia in adult patients. 100% of isolates were susceptible to cefotaxime, ceftazidime, piperacillin-tazobactam, imipenem, ciprofloxacin, gentamicin, and amikacin. 61% were susceptible to cefazolin, 56% to ampicillin, and 33% to fosfomycin. The clinical treatment success rate in patients with bacteremia who received effective empirical antibiotics was as high as 100% [2]. Use of polymyxin B-immobilized fibre column direct hemoperfusion (PMH-DHP) therapy which removes lipopolysaccharide from Gram-negative organisms along with antibiotics was reported to be successful in treating sepsis caused by *P. agglomerans* infection in a patient with small cell carcinoma of the lung [14]. In contrast to the proven pathologic role, some beneficial traits of *P. agglomerans* have been mentioned recently for their role in antibiotic production as an immune potentiator in cancer patients, as a biopesticide and food preservation [15]. The clinical course of the disease in most adult cases is usually mild, unlike the pediatric population; however, it can cause severe illness leading to septicemia, DIC, and multi-organ failure [16]. Application of the proper antibiotic treatment leads to full recovery, even in immunocompromised individuals, as we saw in our patient [2].

The possible cause in our patient might be wound superinfection. Another reason can be gastric mucosal injury leading to translocation of bacteria through the GI tract because of possible GERD, although the patient was not on antacids prior to bacteremia. The patient had DM-I and chronic untreated viral hepatitis which are associated with high rates of spontaneous bacteremia. The clinical course was mild and the patient recovered fully with effective antibiotic treatment without any complication.

## 5. Conclusion

*Pantoea agglomerans* is a pathogen of low virulence even in an immunocompromised adult host, causing diverse clinical picture, and can be treated successfully with proper antibiotic use.
